# Exploring Caregiver Interest in and Preferences for Interventions for Children With Risk of Asthma Exacerbation: Web-Based Survey

**DOI:** 10.2196/46341

**Published:** 2023-08-02

**Authors:** Gabrielle Pogge, David A Fedele, Erika A Waters, Julia Maki, Jean M Hunleth, Sreekala Prabhakaran, Deborah J Bowen, James A Shepperd

**Affiliations:** 1 Department of Psychology University of Florida Gainesville, FL United States; 2 Division of Public Health Sciences Washington University in St Louis St Louis, MO United States; 3 Department of Bioethics and Humanities University of Washington Seattle, WA United States

**Keywords:** asthma, children, caregivers, decision-making, intervention, asthma exacerbations

## Abstract

**Background:**

Maintaining control of asthma symptoms is the cornerstone of asthma treatment guidelines in the United States. However, suboptimal asthma control and asthma exacerbations among young people are common and are associated with many negative outcomes. Interventions to improve asthma control are needed. For such interventions to be successful, it is necessary to understand the types of interventions that are appealing to caregivers of children with different levels of risk of exacerbation.

**Objective:**

This study aimed to evaluate whether caregivers of children with high (vs low) risk of asthma exacerbation show different levels of interest in and preferences for potential intervention programs and delivery methods.

**Methods:**

We contracted with Ipsos to administer a web-based survey to caregivers of children with asthma who were residing in the United States. Caregivers (N=394) reported their interest (1=not at all; 3=a lot) in 9 possible intervention programs and 8 possible intervention delivery methods. Caregivers also indicated their preferences by selecting the 3 intervention programs and 3 delivery methods that “most” interested them. Finally, caregivers completed 2 open-ended questions asking what other resources might be useful for managing their children’s asthma. We classified children as having a high risk of exacerbation if they had an exacerbation in the past 3 months (n=116) and a low risk of exacerbation if otherwise (n=278).

**Results:**

Caregivers reported higher levels of interest in all intervention programs and delivery methods if they cared for a child with a high risk rather than a low risk of exacerbation. However, regardless of the child’s risk status, caregivers expressed the highest levels of interest in programs to increase their child’s self-management skills, to help pay for asthma care, and to work with the school to manage asthma. Caregivers expressed the highest levels of interest in delivery methods that maintained personal control over accessing information (websites, videos, printed materials, and smartphone apps). Caregivers’ preferences were consistent with their interests; programs and delivery methods that were rated as high in interest were also selected as one of the 3 that “most” interested them. Although most caregivers did not provide additional suggestions for the open-ended questions, a few caregivers suggested intervention programs and delivery methods that we had not included (eg, education about avoiding triggers and medication reminders).

**Conclusions:**

Similar interests and preferences among caregivers of children with high and low risk of exacerbation suggest a broad need for support in managing childhood asthma. Providers could help caregivers by directing them toward resources that make asthma care more affordable and by helping their children with asthma self-management. Interventions that accommodate caregivers’ concerns about having personal control over access to asthma information are likely to be more successful than interventions that do not.

## Introduction

### Background

Asthma affects approximately 6% of children aged <18 years in the United States and accounts for >US $81 billion in total costs [1,2]. Maintaining control over asthma symptoms is the cornerstone of national asthma treatment guidelines [3]. Despite the availability of effective asthma treatment, suboptimal pediatric asthma control is common [4] and is burdensome for both caregivers and their children. Low asthma control increases children’s risk of morbidity [3], including emergency department (ED) visits, hospitalizations, and missed school days, and is associated with lower academic achievement [5], limitations in recreational activities, and lower quality of life (QOL) [6]. In addition, caregivers may experience psychological distress about their children’s health [7] and missed work and income when their children need emergency care to treat an acute elevation in symptoms [3,6].

The 2007 National Heart, Lung, and Blood Institute asthma guidelines characterize asthma control in terms of current impairment and future risk of exacerbations (ie, having symptoms so bad that they needed to visit urgent care, ED, or receive a short course of oral steroids) [3]. Research links low asthma control (greater impairment) to greater risk of asthma exacerbation [8-10]. However, even children with well-controlled asthma can experience exacerbations [3,11]. Children who experienced an asthma exacerbation in the last 12 months are at an elevated risk of experiencing a second exacerbation [12,13]. Therefore, these children are most in need of clinical intervention to improve asthma outcomes.

To reduce disease burden and health care costs associated with pediatric asthma, many interventions have focused on increasing asthma control [14]. Interventions are often multifaceted and can include components across multiple levels of asthma care (eg, individual, community, and health care system) such as enabling case management, providing social support, increasing contact and quality of interactions with the health care system, and navigating structural constraints [15,16]. Meta-analyses indicate that these interventions exert small to moderate effects on improving asthma outcomes [17-19].

Recently, the Patient-Centered Outcomes Research Institute and others have led calls for soliciting caregivers’ preferences when designing pediatric asthma interventions [20]. The basic premise is that incorporating such preferences into interventions at early stages will increase the feasibility, acceptability, and effectiveness of the interventions [21]. In the context of asthma, understanding caregivers’ preferences for intervention components and delivery methods may provide important information to increase the uptake and ultimate effectiveness of interventions to improve asthma control [22].

### Objectives

The objective of this study was to identify caregivers’ interests in and preferences for asthma interventions and delivery methods, focusing on how we might develop and deliver future interventions. To accomplish this objective, we developed 3 aims. The first aim was to explore whether caregivers of children who experienced a recent exacerbation (ie, those with a high risk of experiencing future exacerbation) would express greater interest in the intervention programs and delivery methods than would caregivers of children who did not experience a recent exacerbation (ie, those with a low risk of future exacerbation). The second aim was to examine whether the caregivers of children with a high risk of exacerbation differed from the caregivers of children with a low risk of exacerbation in terms of specific intervention programs and delivery methods that they most preferred. Identifying if and how the groups differ in their interest in and preferences for intervention programs and delivery methods can help determine whether it is necessary to tailor interventions to the risk groups and how that tailoring might manifest. Finally, we recognized that caregivers may want interventions that we overlooked. Our third aim was to identify additional programs and delivery methods that caregivers felt would help them control their children’s asthma.

## Methods

### Ethics Approval

The Institutional Review Board at the University of Florida approved all the study procedures and measures (IRB#201802313). All participants provided web-based informed consent. All the study data were anonymized. The details on participant incentives are discussed in the *Design* section.

### Design

We conducted a longitudinal study comprising 2 surveys. Both surveys were administered on the internet to individuals residing in the United States. Data collection for the time 1 (T1) survey was conducted between January 2021 and February 2021. Data collection for the time 2 (T2) survey was conducted 3 months later, between April 2021 and May 2021. Participants received points worth approximately US $20 for completing the T1 survey and approximately US $10 for completing the T2 survey.

### Participants

We aimed to recruit 801 caregivers with complete data at T1. We based our recruitment goal on the sample size needed to test a priori hypotheses related to caregivers’ beliefs about their children’s risk of having an asthma exacerbation [23].

Caregivers were eligible to participate if they reported having a child who (1) was diagnosed with asthma by a health care provider, (2) still had asthma at T1, (3) currently had a prescription for albuterol, (4) was aged <18 years, and (5) resided with the caregiver who would complete the survey for at least 90 days in 1 year. To ensure adequate representation of caregivers with lower income, whose children are especially susceptible to increased asthma morbidity, we stratified recruitment with a goal of no more than one-third of caregivers reporting an income >US $50,000 (unfortunately, we were unable to meet this goal).

We contracted with Ipsos [24] to recruit participants. Ipsos is a global market and public-opinion research company. One of its research tools, KnowledgePanel [24], is a probability-based web-based survey panel comprising 60,000 members. Panelists are recruited through address-based sampling, which involves Ipsos mailing invitations to randomly selected addresses in the United States, with the goal of obtaining a panel that represents the broader US population and includes populations that are often excluded from research (eg, people from minoritized racial or ethnic groups and people with low income). Ipsos provides free internet service and a free web-enabled device to households that want to participate but do not have the technological capability to do so. Despite efforts to increase the representativeness of KnowledgePanel participants, it is important to remember that people who complete web-based surveys may differ in important ways from people who do not complete surveys (eg, time availability, interest, and competing demands). In return for completing the surveys, participants received points that they could redeem for goods or services.

Ipsos advised us that because of stringent eligibility criteria, we would not be able to achieve our required sample size based on only KnowledgePanel participants. Therefore, Ipsos subcontracted with several opt-in panels to supplement recruitment. Multimedia Appendix 1 provides detailed information on our recruitment decisions.

### Procedure

After consenting, caregivers completed all demographic items at T1. They completed all child health outcomes and intervention preference items at T2. Caregivers completed the items pertaining to measures of asthma control and exacerbations (in random order) before completing the items assessing preferences for intervention programs and then preferences for intervention delivery methods. The preregistration, protocol, informed consent form, other study materials, data, codebook, and analysis script are available on the Open Science Framework project page [25].

### Materials

#### Exacerbations

At T2, three items assessed the frequency of asthma exacerbations in the past 3 months (continuing to have asthma symptoms even after the use of quick relief medicine, eg, albuterol through a nebulizer or inhaler; needing to visit the ED or urgent care; and needing an oral steroid pill or liquid medications such as prednisone) using a 5-point scale (1=never in the past 3 months; 5=≥4 times). The items were based on the recommendations from several National Institutes of Health institutes for assessing the components of exacerbations [26]. To determine risk status, we classified a caregiver’s child as high risk if the caregiver selected the response “>1” in any of the 3 items (which indicates that an exacerbation occurred in the past 3 months) and as low risk if the caregiver did not select response “>1” in any of the 3 items.

#### Interest in Intervention Programs

At T2, caregivers reported their interest in 10 potential programs to help manage their children’s asthma (1=not at all interested, 2=a little interested, and 3=very interested). The items were informed by our prior research [27-29] and are consistent with the broader literature, including systematic reviews of pediatric asthma interventions [30-33], systematic reviews of the experiences and needs of caregivers of children with asthma [7], work discussing multilevel strategies for improving the self-management of chronic diseases [34], and research related to challenges in obtaining pediatric care in the United States (eg, transportation, cost, and access to physicians) [35-37]. The items began with the following stem:

In the future, our research team hopes to develop resources to help families manage their child’s asthma. We do not currently have these resources but will use your responses to develop them. The next questions ask about types of help that families of children with asthma might want. How interested are you in the following programs?

A program that: (1) connects you with other families to talk about how different people manage their child’s asthma; (2) works with your child’s school to manage your child’s asthma; (3) assigns a nurse or other healthcare provider to your family to help you with your child’s asthma and asthma medicines; (4) helps you and your child talk with doctors about your child’s asthma; (5) helps you and your child get transportation to doctor appointments or to the pharmacy; (6) helps you get your child’s asthma medicines delivered to your home; (7) helps your child see asthma specialists like an allergist or pulmonologist (lung doctor); (8) helps you cover the costs of your child’s asthma-related medical care; (9) helps your child learn how to manage their asthma on their own; and (10) helps you improve your housing (identifying and fixing things that make your child’s asthma worse, getting a landlord to fix problems in your home, or help with moving to a place that would be better for your child’s asthma).

The last item is in line with other intervention efforts [38]. Although the 10 items were intercorrelated (mean interitem correlation [MIC]=0.57; Cronbach α=.93), we examined the items separately to distinguish between more and less preferred programs. However, for completeness, we also reported on the grand mean of the 10 items for the high-risk and low-risk groups separately.

#### Preferences for Intervention Programs

From the list of 10 intervention programs they rated, caregivers chose up to 3 intervention programs they were “most interested” in.

#### Interest in Intervention Delivery Methods

At T2, caregivers reported their interest in 8 potential intervention delivery methods using the same 3-point scale as the items assessing interest in program types. The items began with the following stem:

We want to learn the most useful ways to make asthma resources available to caregivers in future research studies. If you were a participant in our future research, how interested would you be in receiving additional resources via the following methods?

(1) Printed materials you could read, like a booklet or pamphlet, (2) A website, (3) Videos you could watch, (4) An app that you download for a tablet, computer, or smartphone, (5) Text messages from a nurse or other healthcare provider, (6) A phone call with a nurse or other healthcare provider, (7) A web video call (e.g., Zoom) with a nurse or other healthcare provider, and (8) An in-person appointment with a nurse or other healthcare provider.

Although the items were intercorrelated (MIC=0.56; Cronbach α=.91), we examined the items separately to distinguish between more and less preferred methods. However, for completeness, we also reported on the grand mean of the 8 items for the high-risk and low-risk groups separately.

#### Preferences for Intervention Delivery Methods

From the list of 8 intervention delivery methods, caregivers chose up to 3 methods that they were “most interested” in.

#### Open-Ended Interests and Preferences

At T2, we provided 2 open-ended textboxes where caregivers could describe their interest in additional programs and methods we did not include in the survey. The items read as follows:

Are there other methods to receive resources we have not asked about that would be helpful in managing your child’s asthma? If so, please describe those methods in the box below.

#### Other Measures

At the T2 time point, caregivers responded to a revised version of the 5-item Parent Proxy Asthma Control Test [39]. A total of 4 items asked the caregivers to evaluate their children’s impairment from asthma, daytime symptoms, nighttime symptoms, and albuterol use in the past 4 weeks (range 4-20). The fifth item assessed the caregiver’s subjective judgment of their children’s asthma control (range 1-5). We edited the items to improve clarity and to ensure that the items assessing shortness of breath and albuterol use shared the same response options. Consistent with recent research [23,40], we summed the 4 items assessing symptoms and impairment as a measure of asthma control. We labeled the responses to the fifth item as subjective control. All items used 5-point scales, with higher scores indicating less impairment, fewer daytime and nighttime symptoms, less albuterol use, and better subjective asthma control. We excluded “don’t know” and “choose not to respond” responses to any of the 5 items (asthma control: 15/394, 0.04%; subjective control: 4/394, 0.01%).

Finally, caregivers reported their subjective social status [41], their children’s asthma symptom–free days in the last 14 days (range 0-14), their children’s QOL in the past 3 months (1=poor; 5=excellent), and frequency of inhaled corticosteroid use in the past 3 months (1=never in the past 3 months; 5=more than once per day on average) among children prescribed a controller medicine (high risk: 93/116, 80.2%; low risk: 138/278, 49.6%). We created an aggregate measure of socioeconomic status (SES) from 6 items: (1) household income, (2) subjective financial security, (3) caregiver formal education, (4) employment status, (5) home ownership, and (6) perceived social status. Table 1 shows the means and SDs of perceived social status and the frequencies of the remaining items comprising SES. Because the number of scale points differed across items, we followed the procedures and equations used by Dawes [42] to make all the items 5-point scales before combining the items. We computed the scale mean among caregivers who provided valid responses to at least 3 of the 6 items such that higher scores indicated higher SES (MIC=0.37).

**Table 1:** Caregiver and child demographic and health characteristics by exacerbation risk.

Characteristic		Low exacerbation risk (n=278)	High exacerbation risk (n=116)	Full sample (N=394)
**Caregiver’s gender, n (%)**				
	Men	66 (23.4)	28 (24.1)	94 (23.8)
	Women	212 (76.6)	88 (75.9)	300 (76.1)
	Nonbinary	0 (0)	0 (0)	0 (0)
	Gender nonconforming	0 (0)	0 (0)	0 (0)
**Child’s gender, n (%)**				
	Boy	167 (59.5)	72 (62.1)	239 (60.6)
	Girl	111 (40.5)	43 (37.1)	154 (39.1)
	Nonbinary	0 (0)	1 (0.8)	1 (0.2)
	Gender nonconforming	0 (0)	0 (0)	0 (0)
**Caregiver’s race (could choose more than one), n (%)**				
	Asian	14 (5.2)	6 (5.2)	20 (5.1)
	Black or African American	24 (8.9)	17 (14.6)	41 (10.4)
	American Indian or Alaska Native	0 (0)	1 (0.9)	1 (0.2)
	Native Hawaiian or Pacific Islander	0 (0)	0 (0)	0 (0)
	White	231 (82.8)	90 (77.6)	321 (81.7)
	All others (text response)	8 (3)	2 (1.7)	10 (2.5)
**Child’s race (could choose more than one), n (%)**				
	Asian	19 (5.9)	6 (4.5)	25 (5.5)
	Black or African American	51 (15.9)	25 (18.9)	76 (16.8)
	Native American or Alaskan Native	9 (2.8)	2 (1.5)	11 (2.4)
	Native Hawaiian or Pacific Islander	1 (0.3)	0 (0)	1 (0.2)
	White	229 (71.3)	94 (71.2)	323 (71.3)
	All others (text response)	12 (3.7)	5 (3.8)	17 (3.7)
**Caregiver’s ethnicity, n (%)**				
	Non-Hispanic	253 (91)	98 (84.5)	351 (89.1)
	Hispanic	25 (9)	18 (15.5)	43 (10.9)
**Child’s ethnicity, n (%)**				
	Non-Hispanic	232 (83.4)	85 (73.3)	317 (80.4)
	Hispanic	46 (16.5)	31 (26.7)	77 (19.5)
**Caregiver formal education, n (%)**				
	Less than high school	9 (3.2)	3 (2.6)	12 (3)
	High school graduate, general education development or equivalent	30 (10.8)	26 (22.4)	56 (14.2)
	Some college, no degree	62 (22.3)	25 (21.5)	87 (22.1)
	Associate’s degree	32 (11.5)	11 (9.5)	43 (10.9)
	Bachelor’s degree	79 (28.4)	29 (25)	108 (27.4)
	Postgraduate degree	66 (23.7)	22 (19)	88 (22.3)
**Household income (US $), n (%)**				
	≥25,000	31 (11.2)	23 (20.2)	54 (13.8)
	25,001-50,000	47 (17)	25 (21.9)	72 (18.5)
	50,001-75,000	52 (18.8)	23 (20.2)	75 (19.2)
	75,001-100,000	42 (15.2)	20 (17.5)	62 (15.9)
	≤100,000	104 (37.7)	23 (20.2)	127 (32.6)
**Financial security, n (%)**				
	Cannot make ends meet	16 (5.7)	16 (13.8)	32 (8.1)
	Manage to get by	101 (36.3)	48 (41.4)	149 (37.8)
	Enough money to manage, plus extra	120 (43.2)	43 (37.1)	163 (41.4)
	Money is not a problem	41 (14.7)	9 (7.7)	50 (12.7)
**Home ownership, n (%)**				
	Own home	208 (74.8)	86 (74.1)	294 (75.4)
	Rent home	66 (23.7)	30 (25.9)	96 (24.6)
**Employment status, n (%)**				
	Employed	204 (73.9)	82 (70.7)	286 (72.9)
	Not employed	72 (26.1)	34 (29.3)	106 (27)
Caregiver’s age (years), mean (SD)		44.41 (9.13)	43.06 (10.13)	44.01 (9.44)
Child’s age (years), mean (SD)		11.43 (3.87)	10.83 (4.03)	11.25 (3.92)
Asthma control (range 4=low; 20=high), mean (SD)		18.71^a^ (1.63)	14.92^a^ (3.74)	17.62 (2.98)
Subjective asthma control (1=low; 5=high), mean (SD)		4.42^a^ (0.84)	3.65^a^ (0.90)	4.19 (0.93)
Symptom-free days (0 to 14 d), mean (SD)		10.30^a^ (5.13)	7.32^a^ (4.78)	9.43 (5.21)
Child QOL^b^ (1=poor; 5=excellent), mean (SD)		4.30^a^ (0.74)	3.56^a^ (0.88)	4.08 (0.86)
Frequency of ICS^c^ use (1=never; 5=more than once per day), mean (SD)		2.82 (1.37)	2.75 (1.14)	2.79 (1.28)
Subjective social status (1=worst off; 10=best off), mean (SD)		5.88 (1.83)	5.33 (2.26)	5.74 (1.96)
SES^d^ (1=low; 5=high), mean (SD)		3.58^a^ (0.91)	3.33^a^ (0.99)	3.51 (0.94)

^a^Means differ at *P*<.05.

^b^QOL: quality of life.

^c^ICS: inhaled corticosteroid.

^d^SES: socioeconomic status.

### Data Analysis

For descriptive purposes, we first explored demographic differences between the high-risk and low-risk groups by computing correlations between the high-risk and low-risk groups, child health indicators, and SES, along with the scale measures of caregivers’ interest in intervention programs and delivery methods. Second, we examined the item-level and scale mean differences in interest between the high-risk and low-risk groups using independent sample *t* tests (2-tailed). We adjusted the tests and *df* for unequal variance. Third, we computed preferences as the percentage of caregivers in the high-risk and low-risk groups who chose each intervention program and method as “most” interesting and the percentage of caregivers who did not select “most interested” for any of the programs or methods (using *z* test of equal proportions). Finally, we analyzed the open-ended text responses using an inductive approach, identifying categories in the data, in line with recommendations for qualitative analysis [43]. We reviewed the responses for patterns and then grouped the responses based on common themes (eg, education and financial resources). We then coded the responses according to these categories and summarized the content within each category to describe the range of responses.

## Results

### Sample Characteristics

In total, 888 caregivers of children with asthma completed the T1 survey. Owing to attrition, only 401 caregivers completed the T2 survey (401/888, 45.2%), which included the measures of intervention preferences. The final sample size for analysis was 394; a total of 116 (29.4%) children were considered at high risk of exacerbation, and 278 (70.6%) were considered at low risk (we had inadequate information to classify 7 responses as low or high risk). We also computed the average of the 3 exacerbation items in the high-risk group for descriptive purposes (mean 2.07, SD 0.87).

Overall sample characteristics are listed in Table 1. Notably, although the distribution of race and ethnicity roughly resembles that of the broader US population, our sample includes people with more formal education and higher income. As evident in Table 2, caregivers of children at high risk for an exacerbation reported worse asthma control and subjective asthma control, fewer symptom-free days, and poorer child QOL than did caregivers of children at low risk. The means at the bottom of Table 1 reveal the same effects. In addition, the mean education, income, and financial security (as well as the aggregate measure of SES) were lower in the high-risk group than in the low-risk group. Children in the high-risk group were more likely to be Hispanic compared with children in the low-risk group (*χ*^2^_1_=4.8, *P*=.03). Finally, higher scores on the scale measures of interest in intervention programs and delivery methods corresponded with worse child health outcomes and more frequent inhaled corticosteroid use (Table 2).

**Table 2:** Correlations between risk groups, child health outcomes, and socioeconomic status^a^.

Variable			1		2		3		4		5		6		7		8		9
**1. Exacerbation risk**																			
	*r*	1		−0.58		−0.38		−0.26		−0.40		−0.03		−0.12		0.34		0.32	
	*P* value	—^b^		<.001		<.001		<.001		<.001		.70		.02		<.001		<.001	
**2. Asthma control**																			
	*r*	−0.58		1		0.40		0.34		0.46		−0.15		0.15		−0.44		−0.41	
	*P* value	<.001		—		<.001		<.001		<.001		.03		.003		<.001		<.001	
**3. Subjective control**																			
	*r*	−0.38		0.40		1		0.34		0.41		0.09		0.21		−0.17		−0.12	
	*P* value	<.001		<.001		—		<.001		<.001		.17		<.001		<.001		.02	
**4. Symptom-free days**																			
	*r*	−0.26		0.34		0.34		1		0.34		0.03		0.22		−0.11		−0.14	
	*P* value	<.001		<.001		<.001		—		<.001		.67		<.001		.02		.005	
**5. Child QOL^c^**																			
	*r*	−0.40		0.46		0.41		0.34		1		0.02		0.23		−0.31		−0.29	
	*P* value	<.001		<.001		<.001		<.001		—		<.001		<.001		<.001		<.001	
**6. ICS^d^ use**																			
	*r*	−0.03		−0.15		0.09		0.03		0.02		1		0.04		0.14		0.13	
	*P* value	.70		.03		.17		.67		.70		—		.59		.07		.05	
**7. SES^e^**																			
	*r*	−0.12		0.15		0.21		0.22		0.23		0.04		1		−0.04		−0.08	
	*P* value	.02		.003		<.001		<.001		<.001		.59		—		.25		.10	
**8. Interest in intervention programs**																			
	*r*	0.34		−0.44		−0.17		−0.11		−0.31		0.14		−0.04		1		0.78	
	*P* value	<.001		<.001		<.001		.02		<.001		.04		.25		—		<.001	
**9. Interest in delivery methods**																			
	*r*	0.32		−0.41		−0.12		−0.14		−0.29		0.13		−0.08		0.78		1	
	*P* value	<.001		<.001		.02		.005		<.001		.04		.10		<.001		—	

^a^For exacerbation risk, 1=high risk and 0=low risk. For all other measures, higher scores indicate more constructs assessed.

^b^Not applicable.

^c^QOL: quality of life.

^d^ICS: inhaled corticosteroid.

^e^SES: socioeconomic status.

### Interest in and Preferences for Intervention Programs

As evident in Table 3, the level of interest in each of the programs was higher on average in the high-risk group than in the low-risk group (*P*<.001 for all programs), as was the overall scale measure of interest in programs. In addition, regardless of their children’s risk, caregivers expressed the greatest interest in programs that would help their children with asthma self-management, help pay for asthma care, and help work with the child’s school to manage their child’s asthma. They expressed the lowest interest in having a health care manager, connecting with other families about managing the child's asthma, and helping with transportation.

A similar pattern emerged for preferences (ie, when caregivers selected up to 3 intervention programs they were “most interested” in). Specifically, a higher percentage of caregivers indicated that they preferred programs related to children’s asthma self-management skills and financing asthma care, and a lower percentage of caregivers indicated that they preferred programs to help with transportation, connect with other families to discuss asthma care, and have a personal health care manager. However, the percentage of caregivers who did not prefer any of the programs (ie, they did not select “most interested” for any of the programs) was higher for caregivers of children with low risk of exacerbation (60/278, 21.6%) than high risk of exacerbation (12/116, 10.3%; *χ*^2^_1_=6.2, *P*=.01).

**Table 3:** Interest in and preferences for intervention programs^a^.

			Outcome												
			Interest										Preferences^b^		
			Exacerbation risk					Test statistics					Exacerbation risk		
			Low (n=278), mean (SD)		High (n=116), mean (SD)		*t* test (*df*)			M_diff_ 95% CI^c^		Low (n=278), n (%)			High (n=116), n (%)
**Intervention program**															
	Asthma self-management	1.91 (0.80)		2.27 (0.75)		4.32 (227.59)			0.20-0.53		125 (45)			48 (41.4)	
	Financial help	1.83 (0.85)		2.21 (0.81)		4.18 (226.18)			0.20-0.56		106 (38.1)			42 (36.2)	
	Work with school to manage asthma	1.71 (0.71)		2.11 (0.79)		4.72 (196.20)			0.23-0.57		65 (23.4)			33 (28.4)	
	Connect with asthma or allergy specialist	1.58 (0.71)		2.11 (0.83)		6.03 (189.47)			0.36-0.71		58 (20.9)			34 (29.3)	
	Medicine home delivery	1.60 (0.76)		2.08 (0.83)		5.33 (198.74)			0.30-0.66		75 (27)			29 (25)	
	Fix housing problems	1.56 (0.73)		2.07 (0.84)		5.62 (189.05)			0.33-0.69		N/A^c^			N/A	
	Help talking with doctors about asthma	1.46 (0.67)		1.97 (0.81)		5.98 (183.99)			0.34-0.68		26 (9.3)			23 (19.8)	
	Health care manager to help with asthma	1.43 (0.64)		1.90 (0.83)		5.46 (174.28)			0.30-0.64		23 (8.3)			23 (19.8)	
	Connect with other families about managing child’s asthma	1.44 (0.60)		1.87 (0.76)		5.43 (177.47)			0.27-0.59		24 (8.6)			18 (15.5)	
	Transportation	1.19 (0.49)		1.62 (0.82)		5.31 (150.32)			0.27-0.59		11 (3.9)			13 (11.2)	
Overall scale measure			1.57 (0.53)		2.02 (0.64)		6.62 (179.15)			0.32-0.58		N/A			N/A

^a^The response scale ranged from 1 (not at all interested) to 3 (very interested). For all rows, the group means differed using independent *t* tests at *P*<.001.

^b^Responses to an item asking caregivers to select up to 3 programs they were most interested in.

^c^N/A: not applicable; because of a survey programming error, this item was not included in the question asking about participant preferences (ie, choose the 3 methods they were “most interested” in).

### Interest in and Preferences for Intervention Delivery Methods

As evident in Table 4, the level of interest in each of the delivery methods was higher on average in the high-risk group than in the low-risk group (*P*<.001 for all delivery methods), as was the overall scale measure of interest in delivery methods. Regardless of their children’s risk, caregivers expressed the highest interest in receiving additional resources via websites, videos, or printed materials. They expressed the lowest interest in text messages from providers, phone calls with providers, web video calls with providers, and in-person appointments with health care providers.

A similar pattern emerged for delivery method preferences as for interest; a higher percentage of caregivers indicated that they preferred receiving additional resources via websites, videos, or printed materials, and a lower percentage of caregivers preferred receiving additional resources via interactions with health care providers. In contrast to the intervention program preferences, child risk was not related to preferences in intervention delivery methods. Instead, similar proportions of caregivers of children with low risk of exacerbation (58/278, 20.9%) and caregivers of children with high risk of exacerbation (19/116, 16.4%; *χ*^2^_1_=0.8, *P*=.38) preferred none of the methods (ie, they did not select “most interested” for any of the methods).

**Table 4:** Interest in and preferences for intervention delivery methods^a^.

			Outcome											
			Interest									Preferences^b^		
			Exacerbation risk				Test statistics					Exacerbation risk		
			Low (n=278), mean (SD)		High (n=116), mean (SD)		*t* test (*df*)	M_diff_ 95% CI				Low (n=278), n (%)		High (n=116), n (%)
**Delivery methods**														
	Website	1.99 (0.73)		2.31 (0.70)		4.09 (222.58)			0.17-0.47		144 (51.8)		52 (44.8)	
	Videos	1.74 (0.73)		2.15 (0.80)		4.80 (200.27)			0.24-0.58		80 (28.8)		35 (30.2)	
	Printed materials (eg, pamphlet)	1.75 (0.72)		2.12 (0.75)		4.50 (209.69)			0.21-0.53		107 (38.5)		41 (35.3)	
	App (eg, for tablet or smartphone)	1.62 (0.69)		2.12 (0.72)		6.30 (207.33)			0.34-0.65		63 (22.7)		39 (33.6)	
	SMS text messages from provider	1.51 (0.66)		1.91 (0.81)		4.80 (181.37)			0.24-0.57		23 (8.3)		21 (18.1)	
	In-person appointment with provider	1.52 (0.65)		1.89 (0.85)		4.19 (173.54)			0.19-0.54		43 (15.5)		22 (19)	
	Web video call with provider	1.40 (0.61)		1.82 (0.84)		4.83 (167.67)			0.24-0.59		27 (9.7)		25 (21.5)	
	Phone call with provider	1.37 (0.60)		1.79 (0.83)		4.97 (168.05)			0.25-0.59		22 (7.9)		15 (12.9)	
Overall scale measure			1.61 (0.50)		2.01 (0.63)		6.07 (178.11)			0.27-0.53		N/A^c^		N/A

^a^The response scale ranged from 1 (not at all interested) to 3 (very interested). For all rows, the group means differ using independent *t* tests at *P*<.001.

^b^Responses to an item asking caregivers to select up to 3 programs they were most interested in.

^c^N/A: not applicable.

### Open-Ended Responses

Most caregivers left the open-ended response boxes empty or merely said they had no additional suggestions. Many of the responses added were elaborations on the programs and methods we stated. However, a few caregivers suggested programs and delivery methods that were not included. Regarding intervention programs, the most common suggestion was for education programs on topics such as identifying and avoiding triggers at home and elsewhere, identifying symptoms, administering medications, knowing possible alternative treatments such as meditation and alternative medicines, understanding insurers, and understanding treatments. Caregivers suggested education programs tailored for their children or for others in their children’s life (eg, coaches).

Caregivers also suggested tools (perhaps documents, web pages, or apps) that could help manage their child’s asthma. Suggestions included a checklist of asthma symptoms, decision tree on how to respond to symptoms, reminders on how to administer medications, procedure for managing medicines and refills, and log for tracking symptoms. Finally, caregivers suggested child peer-support groups and a local map to identify safe and unsafe indoor and outdoor places for a child with asthma.

Regarding the delivery method, caregivers provided 4 suggestions that were not covered in our survey, and all the 4 centered on education. They included visually engaging classes or printed educational materials and games for children that subtly provided information about asthma.

## Discussion

### Principal Findings

Our research had three aims: to examine whether (1) interest in and (2) preferences for intervention programs and delivery methods among caregivers of children with high risk of exacerbation differed from those among caregivers of children with low risk of exacerbation and (3) to explore whether caregivers had intervention and delivery preferences that researchers have overlooked.

For aim 1, our results revealed that caregivers of children with a high risk of exacerbation expressed greater interest for all intervention programs and delivery methods than caregivers of children with a low risk of exacerbation. In addition, children’s risk of exacerbation was not associated with caregivers’ interest in specific programs or delivery methods; regardless of the exacerbation risk, programs that helped children self-manage asthma and manage asthma care costs and delivery methods that maintained caregivers’ personal control over accessing information (eg, websites, videos, or printed materials) received the highest mean interest ratings.

For aim 2, the order of preferences for the programs and delivery methods (ie, those selected as caregivers’ top 3 preferences) was generally similar for caregivers of children with high risk and those with low risk of exacerbation (but note that caregivers of children with high exacerbation risk expressed stronger preferences for programs to help work with schools to manage asthma and access an asthma specialist than caregivers of children with low exacerbation risk). Furthermore, the specific intervention programs and delivery methods that caregivers preferred were the same programs and methods that they were interested in.

Finally, for aim 3, many of the open-ended responses echoed the findings from the item ratings. In addition, caregivers suggested programs that provided education, helped track symptoms, and provided peer support to their children with asthma. They also suggested delivery methods such as classes and computer games that included information about asthma for their children.

### Interpretations, Implications, and Comparison With Existing Literature

Effective self-management is the treatment goal for all people with asthma [11]. Education programs to facilitate self-management can be cost effective and can reduce children’s school absences, ED visits, and days with restricted activity, especially among children with high risk of exacerbation [19]. Although we did not ask whether caregivers preferred interventions delivered in schools, caregivers did express interest in programs that facilitated working with the school to manage children’s asthma. Children spend large amounts of time in school, and school nurses can provide education, advice, and direct care that empowers children and their caregivers to improve asthma management [30]. Thus, it seems reasonable to develop strategies for disseminating and implementing interventions aimed at improving asthma management in children while they are in school [44].

Comprehensive asthma programs that provide case management services for asthma-related medical care, home visits, and environmental assessments and remediation have demonstrated reductions in hospitalizations and ED visits in high-risk pediatric populations [45]. However, desire for a personal health care manager fell toward the bottom of the caregivers’ interests and preferences. It is possible that the limited description of a “health care manager” in our survey item (ie, “assigns a nurse or other health care provider to your family to help you with your child’s asthma and asthma medicines”) did not adequately capture the potential benefits of such programs. It could also be that caregivers preferred not to allow a stranger into their private space. The lack of efficient and reliable transportation can also be a barrier to asthma management, particularly in rural areas [46] (but see the study by Lee et al [47] for information about the normalization of long travel distances in rural areas). Because our sample of caregivers was, on average, well resourced, it is perhaps not surprising that transportation was a less preferred program.

The intervention programs and delivery methods that caregivers rated the highest suggest several ways in which providers can assist caregivers and children. First, even though the sample was overrepresented by caregivers with high household income, interest in financial programs was high. One possible implication of this interest is that providers could assist caregivers, particularly those who struggle financially, by directing them toward resources that make asthma care more affordable. Second, interest in asthma self-management suggests that providers could offer caregivers and their children guidance on how children can self-manage their asthma. However, any such guidance should be sensitive and responsive to the structural, social, and individual barriers that can undermine their child’s self-management [48]. Third, the open-ended responses suggest that caregivers wanted interventions that provided asthma education, helped them track their children’s symptoms, and provided peer support for their children with asthma. Providers are well positioned to help with these needs. For example, providers can facilitate the development of peer-support groups for children with asthma, where children can share their experiences and knowledge of asthma. Finally, the ratings of the delivery methods suggest that caregivers favor methods that allow personal control over accessing information. These findings are consistent with the burgeoning literature indicating family preferences for eHealth- or technology-based interventions [49]. Providers could share information sources that caregivers can access on their own and without the involvement of health care providers.

### Limitations

Our decision to categorize participants based on recent exacerbation rather than on asthma control was based on the high overlap between asthma control and exacerbations [9], psychometric concerns about assessing asthma control via survey measures [40,50], and evidence that caregivers appear to conflate low asthma control with the experience of an asthma exacerbation [29,40]. Nevertheless, it would be useful to examine whether our findings replicate objective measures of asthma control such as spirometry results. In addition, despite our efforts to recruit participants with low income, participants with high income were overrepresented in our sample, and this overrepresentation likely influenced our results (eg, possibly by lowering the importance of transportation support). We had limited space in the survey to describe the programs and methods that we presented to caregivers. Had we described the programs and methods in greater detail, it is possible that the caregivers would have responded differently. Relatedly, we did not offer caregivers the option to express interest in programs that provided psychosocial support. Finally, the survey company we used provides computers and free internet services to panel members who cannot afford them. However, it is likely that web-based surveys underrepresent people with low digital literacy, and their preferred methods of intervention delivery may have differed, leaning more toward delivery methods that are print, audio, and video based.

### Conclusions

The take-home message of our study is clear: with limited exceptions, caregivers of children with high risk and low risk of asthma exacerbation do not generally differ in the intervention programs that they prefer. Instead, they differ in the magnitude of their interest. Specifically, caregivers of children with a high exacerbation risk reported greater interest in all intervention programs and delivery methods. Further investigation revealed that regardless of the risk status, caregivers most preferred programs that increased their children’s self-management ability, helped pay for asthma care, and worked with the children’s school to manage asthma. In addition, the open-ended responses revealed a strong interest in greater asthma education, peer groups for their children, and ways to log and manage their children’s asthma. Finally, caregivers expressed the greatest interest in delivery methods that maintained personal control over accessing information without the need for involvement from a health care provider (websites, videos, and printed materials).

The fact that the preferred intervention programs, particularly those that provided financial assistance for asthma-related expenses, were the same in both high- and low-risk groups may be a statement about the inadequacy of support provided in the United States for children with chronic health problems. Caring for a child with a chronic health problem can be expensive and complicated [7], and government agencies in the United States do less than other high-income nations to support children and their caregivers [51]. Furthermore, caregivers’ relative disinterest in programs that entailed contact with health care providers or other caregivers, and their interest in delivery methods that were accessible in their homes, may indicate that caregivers believe they already have sufficient access to health care providers. However, considering the literature demonstrating some caregivers’ feelings of alienation from providers [28], it is also reasonable to hypothesize that caregivers may prioritize other avenues of help.

Caregivers’ interest in helping their children learn self-management may have several origins. First, caregivers may seek to overcome inadequate institutional, neighborhood, or interpersonal support for a child’s asthma care by teaching their children how to avoid triggers, manage symptoms, and advocate for themselves [48,52]. Second, caregivers may want a respite from the stress of caring for their children’s asthma [7]. Finally, caregivers may want assistance in facilitating the transition to independence as the child matures and becomes more involved in their own asthma self-management [48]. Regardless of the origin, these data suggest unmet needs among caregivers.

Our findings provide insights into the interventions and delivery methods that caregivers prefer and suggest that interventionists may not need to tailor the types of programs or delivery methods based on children’s risk of exacerbation. It also suggests that regardless of exacerbation risk, caregivers prioritize delivery methods that enable privacy and control. The next step for researchers is to find ways to translate these findings into programs that can benefit caregivers.

## Appendix

Multimedia Appendix 1 Detailed description of recruitment and data quality decisions.

## Abbreviations

ED: emergency department
MIC: mean interitem correlation
SES: socioeconomic status
T1: time 1
T2: time 2

## Data Availability

The data sets generated during and analyzed during this study are available in the Open Science Framework repository [25].
