# Supplementary material for: Exploring Caregiver Interest in and Preferences for Interventions for Children With Risk of Asthma Exacerbation: Web-Based Survey
Source: JMIR Form Res. 2023 Aug 2;7:e46341. doi: 10.2196/46341 (PMC10433025; doi:10.2196/46341)
Supplement: Multimedia Appendix 1 [file formative_v7i1e46341_app1.docx]

**Exploring Caregiver Interest in and Preferences for Interventions on Child Asthma Exacerbation Risk: A Preliminary User Needs Analysis**

**Online Supplemental Materials**

**Detailed description of recruitment and data quality decisions**

Ipsos maintains online panels of individuals who agree to be contacted for marketing or academic research. When individuals register with Ipsos, they complete a questionnaire assessing standard socio-demographic characteristics, household characteristics, and health conditions. Ipsos invited 26,118 individuals aged 18 and older to participate in this study. All of the individuals indicated on their Ipsos intake survey that they had a child under age 18. Of those, 3,857 were members of their English-language participant database *KnowledgePanel*, and 22,261 were from outside opt-in panels. Of the 26,118 individuals invited, 6154 clicked the survey link and 4480 were screened out for the following reasons: 4208 (68.4% of the 6154 people who clicked the link) because they did not report having a child with asthma, 141 (2.3%) because the child no longer had asthma, 17 (0.3%) because the child was 18 years or older, 52 (0.8%) because the child did not have a prescription for albuterol, and 62 (1.0%) because the child did not live with the caregiver at least 90 days a year. An additional 786 (12.8%) respondents exited the survey before completing it. The final sample included 888 (14.4% of total invitations) caregivers who qualified and completed the survey. Of those, 417 (46.9%) were from KnowledgePanel and 471 (53.0%) were from the opt-in panels.

We conducted a limited survey launch involving 149 participants to check that the median survey duration was no longer than 30 minutes. Because the survey length among the soft launch participants was about 9 minutes too long, we dropped 45 items we had already flagged for possible deletion and performed scale construction analyses on the behavior items to help us decide if we could drop other items. The items we dropped after soft launch are identified in the ‘Procedure’ table in the pre-registration document (<https://osf.io/xzy8e/?view_only=cb943774900946c1939889a57cccec0c>). We posted the final pre-registration prior to any analysis of our hypotheses.

Of the 888 individuals who were eligible for and completed the survey, we omitted from analysis data from 74 (8.3%) respondents who failed one or both of two data quality indicators: (a) speeding (completing the survey in less than half of the median survey completion time of 33 minutes) and (b) low self-reported attention (“not at all” or “a little”, or “choose not to respond”) based on the response to an item at the end of the survey that asked respondents how much they were paying attention during the survey.

The final sample comprised 814 participants, of which 405 were Ipsos Knowledge Panel members and 409 were opt-in panel members recruited by Ipsos from other national survey panels. Participants were compensated for their time based on Ipsos’ recruitment process, which includes modest incentives for ongoing participation in their survey panel and study-specific incentives. Ipsos handled all participant compensation.
